# Cardiac inotropy, lusitropy, and Ca^2+^ handling with major metabolic substrates in rat heart

**DOI:** 10.1007/s00424-016-1892-8

**Published:** 2016-10-28

**Authors:** Zai Hao Zhao, Jae Boum Youm, Yue Wang, Jeong Hoon Lee, Jae Hwi Sung, Joon-Chul Kim, Sun Hee Woo, Chae Hun Leem, Sung Joon Kim, Lan Cui, Yin Hua Zhang

**Affiliations:** 1 Department of Physiology & Biomedical Sciences, Ischemic/hypoxic disease institute, Seoul National University, College of Medicine, 103 Dae Hak Ro, Jongno Gu,, Seoul, 110-799 South Korea; 2 Department of Physiology, College of Medicine, Inje University, Busan, South Korea; 3 College of Medicine/Asan Medical Center, University of Ulsan, Ulsan, South Korea; 4 College of Pharmacy, Chungnam National University, Daejeon, South Korea; 5 Yan Bian University Hospital, Yanji, Ji Lin China; 6 Division of Cardiovascular Sciences, Manchester University, Manchester, UK

**Keywords:** Cardiac myocyte, Contraction, Intracellular Ca^2+^ transient, Metabolic substrates, Myofilament Ca^2+^ sensitivity, pH, Relaxation

## Abstract

Fatty acid (FA)-dependent oxidation is the predominant process for energy supply in normal heart. Impaired FA metabolism and metabolic insufficiency underlie the failing of the myocardium. So far, FA metabolism in normal cardiac physiology and heart failure remains undetermined. Here, we evaluate the mechanisms of FA and major metabolic substrates (termed NF) on the contraction, relaxation, and Ca^2+^ handling in rat left ventricular (LV) myocytes. Our results showed that NF significantly increased myocyte contraction and facilitated relaxation. Moreover, NF increased the amplitudes of diastolic and systolic Ca^2+^ transients ([Ca^2+^]_i_), abbreviated time constant of [Ca^2+^]_i_ decay (tau), and prolonged the peak duration of [Ca^2+^]_i_. Whole-cell patch-clamp experiments revealed that NF increased Ca^2+^ influx via L-type Ca^2+^ channels (LTCC, I_Ca-integral_) and prolonged the action potential duration (APD). Further analysis revealed that NF shifted the relaxation phase of sarcomere lengthening vs. [Ca^2+^]_i_ trajectory to the right and increased [Ca^2+^]_i_ for 50 % of sarcomere relengthening (EC_50_), suggesting myofilament Ca^2+^ desensitization. Butanedione monoxime (BDM), a myosin ATPase inhibitor that reduces myofilament Ca^2+^ sensitivity, abolished the NF-induced enhancement of [Ca^2+^]_i_ amplitude and the tau of [Ca^2+^]_i_ decay, indicating the association of myofilament Ca^2+^ desensitization with the changes in [Ca^2+^]_i_ profile in NF. NF reduced intracellular pH ([pH_i_]). Increasing [pH]_i_ buffer capacity with HCO_3_/CO_2_ attenuated Δ [pH]_i_ and reversed myofilament Ca^2+^ desensitization and Ca^2+^ handling in NF. Collectively, greater Ca^2+^ influx through LTCCs and myofilament Ca^2+^ desensitization, via reducing [pH]_i_, are likely responsible for the positive inotropic and lusitropic effects of NF. Computer simulation recapitulated the effects of NF.

**Electronic supplementary material:**

The online version of this article (doi:10.1007/s00424-016-1892-8) contains supplementary material, which is available to authorized users.

The heart is an *omnivorous* organ that relies on both fatty acids and carbohydrates to produce myocardial ATP. Constant supply of ATP through cardiac metabolism is essential to maintain the normal functions of the heart [15, 20]. In healthy heart, fatty acid-dependent beta-oxidation accounts for 70–90 % of myocardial ATP and the remaining amount is produced through carbohydrate metabolism (such as glucose oxidation and glycolysis) [15]. However, during disease progression (hypertrophy and early stage heart failure), glucose oxidation and glycolysis become the predominant sources of cellular ATP, indicating a *metabolic shift* from fatty acids to glucose utilization [15]. At the end stage of heart failure, both glucose metabolism and beta-oxidation are dysregulated, resulting in energy deficiency and impaired myocardial contraction [15]. Until recently, glucose has been used as the *sole* metabolic substrate in most of cardiac physiological studies (despite the importance of fatty acids in cardiac metabolism). Accordingly, we aim to evaluate the impact of metabolic substrates’ supplementation on the mechanisms mediating cardiac contractility in normal rat hearts.

Excitation–contraction (E–C) coupling is the main scheme for the interpretation of myocyte contraction, where Ca^2+^ handling plays a central role to activate the event [2, 8]. Initially, Ca^2+^ influx through voltage-dependent L-type Ca^2+^ channels (LTCC, I_Ca-L_) triggers more Ca^2+^ to release from the sarcoplasmic reticulum (SR) via ryanodine receptors (RyR). Intracellular Ca^2+^ ([Ca^2+^]_i_) binds to troponin C (TnC) in the thin filament (troponin complex), prompts actin association with myosin, and initiates contraction. Reuptake of Ca^2+^ into the SR via Ca^2+^-dependent ATPase (SERCA) and Ca^2+^ extrusion via Na^+^-Ca^2+^ exchanger (NCX) decrease intracellular Ca^2+^, lead to the dissociation of Ca^2+^ from TnC and myosin from actin, myofibril relaxes. As such, factors that increase or decrease the amount of intracellular Ca^2+^ plays important roles in myocardial contractile function. In addition, myofilament Ca^2+^ sensitivity is pivotal in determining myocyte contraction and relaxation in the functioning heart. On the other hand, recent consensus is that changes in the myofilament Ca^2+^ sensitivity, per se, regulates intracellular Ca^2+^ homeostasis and therefore myocyte contractility in normal and diseased hearts [4, 11, 19, 21]. Until recently, properties of intracellular Ca^2+^ handling and myofilament Ca^2+^ sensitivity in mediating metabolic substrates’ regulation of cardiac contractile function has not been reported yet.

Here, we aim to analyze the effects of metabolic substrate supplementation (fatty acids: palmitic acid, linoleic acid, and oleic acid; glucose; pyruvate; and lactate, termed *nutrition with fatty acids*, NF) on myocyte contraction, relaxation, intracellular Ca^2+^ handling, and myofilament Ca^2+^ sensitivity. Our results have shown that myocyte contraction and intracellular Ca^2+^ transients are enhanced and relaxation is facilitated by NF. Increased Ca^2+^ influx via LTCC and, counterintuitively, myofilament Ca^2+^ desensitization by reduced intracellular pH are involved in the effects of NF in rat cardiac myocytes.

## Methods and materials

### Animals and isolation of LV myocytes

Sprague–Dawley rats (12 weeks old, male) were anesthetized with pentobarbital sodium (30 mg/kg, i.p.), and the hearts were extracted and rapidly mounted onto the Langendorff perfusion system which were then perfused with a nominal Ca^2+^-free solution for 10 min (in mM; NaCl 135, KCl 5.4, MgCl_2_ 3.5, glucose 5, HEPES 5, Na_2_HPO_4_ 0.4, taurine 20 at pH of 7.4, NaOH), followed by a further 8-min perfusion with the same solution containing collagenase (1 mg/ml, Worthington Biochemical Co.; protease, 0.133 mg/ml, BSA 1.65 mg/ml; Ca^2+^ 0.05 mM). Afterward, the LV free wall was dissected and incubated in fresh collagenase-only solution. Myocytes were harvested following a further 10-min digestion period, washed and resuspended in storage solution (in mM; NaCl 120, KCl 5.4, MgSO_4_ 5, CaCl_2_ 0.2, Na-pyruvate 5, glucose 5.5, taurine 20, HEPES 10, mannitol 29, pH 7.4, NaOH). The myocyte suspension was stored at room temperature and cells were used within 8 h of isolation. The study protocol was in accordance with the Guide for the Care and Use of Laboratory Animals published by the US National Institutes of Health (NIH Publication No. 85-23, revised 1996). It is approved by the Institutional Animal Care and Use Committee (IACUC) in Seoul National University (IACUC approval no.: SNU-101213-1).

**Scheme 1:**
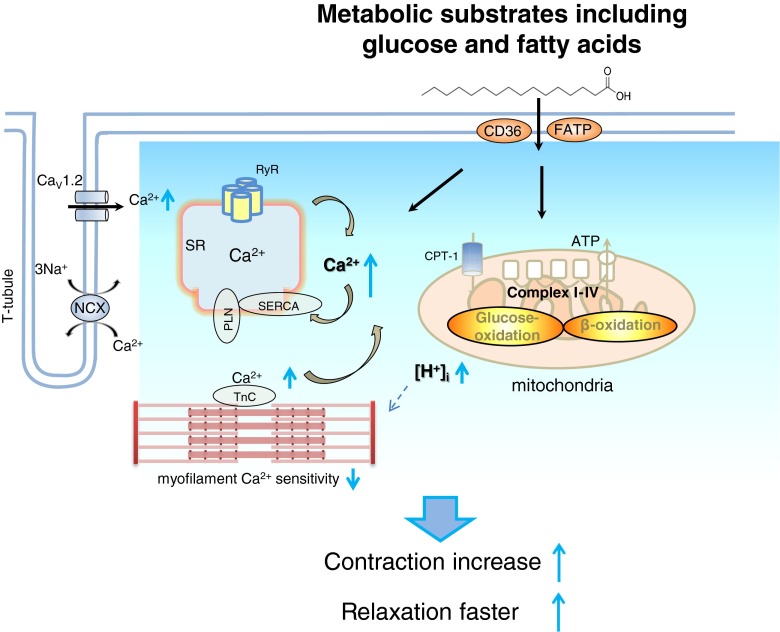
Schematic diagram of cardiac excitation–contraction coupling in the presence of major metabolic substrates including fatty acids and glucose at physiological concentration. Contraction is enhanced with metabolic substrates’ supplementation. Mechanistically, Ca^2+^ influx through LTCC is increased, leads to prolonged APD and greater Ca^2+^ release from the SR, and increases intracellular free Ca^2+^. Reuptake of Ca^2+^ via SERCA is facilitated. Myofilament Ca^2+^ sensitivity is reduced, possibly via reduced pH_i_, which in part is responsible for greater intracellular Ca^2+^ level and decline in rat LV myocytes

### Chemical compositions in NT and “nutrition with fatty acids-NF” solution

Metabolic substrates (oleic acid 200 μM, palmitic acid 100 μM, linoleic acid 100 μM, lactate 1 mM, pyruvate 100 μM, and carnitine 50 μM) were supplemented to the normal Tyrode solution (NT, in mM: NaCl 141.4, KCl 4, NaH_2_PO_4_ 0.33, MgCl_2_ 1, HEPES 10, glucose 5.5, CaCl_2_ 1.8). Fatty acids (oleic acid, palmitic acid, linoleic acid) were dissolved in 1.35 mM (0.09 g/ml) BSA in NT solution at 70 °C for 3 h to make respective stock solutions (10 mM). The composition and the concentrations of metabolic substrates are modified from blood sample results of normal rats [14].

### Measurement of contraction and Ca^2+^ transients

Changes in sarcomere length and Ca^2+^ transients were measured in LV myocytes by using a video-sarcomere detection system (IonOptix Corp). For Ca^2+^ measurements, LV myocytes were preincubated with a Ca^2+^ indicator, acetoxymethyl ester of Fura-2 AM (2 μM) in perfusion solution containing 250 μM Ca^2+^ for 15 min at room temperature in the dark. LV myocytes were then washed in perfusion solution containing 500 μM Ca^2+^ for 10 min. The Fura-2 loaded LV myocytes were kept in perfusion solution (500 μM Ca^2+^) before being used. Measurements from at least 10 steady-state contractions were averaged for each myocyte and for each stage of the experimental protocols. All experiments were carried out at 36 ± 1 °C and field stimulated at 2 Hz.

### Measurement of myofilament Ca^2+^ sensitivity

Sarcomere length and Fura-2 ratio (F_360_/F_380_) were recorded simultaneously (field stimulation, 2 Hz). The phase-plane loop of the Fura-2 ratio vs. sarcomere shortening of the same myocyte was plotted and the data were averaged for each group. In each plot, define Fura-2 ratio at 50 % relaxation (EC_50_). Compare both the loop and EC_50_ of each intervention.

### Measurement of L-type Ca^2+^ current using whole-cell voltage clamp technique

L-type Ca^2+^ current (I_Ca_) was measured with whole-cell voltage clamp technique (Axon instrument, 200 A) in LV myocytes in NT and in NF. I_Ca_ was elicited by 200-ms step depolarization protocol from a holding potential of −40 mV to test potentials from −60 mV to +40 mV. Current–voltage relationship, peak current density (pA/pF at 0 mV), and I_Ca_ integral at −20, 0, and +20 mV for 50 ms from the I_Ca_ peak were analyzed. Pipette solution for I_Ca_ measurement contained (in mM) 120 K aspartate, 20 KCl, 10 HEPES, 5 MgATP, 10 NaCl, pH adjusted to 7.2 using KOH.

### Measurement of action potential using current clamp technique

The action potential profile of the myocyte was recorded with current clamp technique in NT and in NF (sampling rate: 10 kHz) at 35–36 °C (Axon Instruments, 200 A). The pipette (resistance 1–2 MΩ) solution contains (in mM) 110 K-aspartate, 30 KCl, 5 NaCl, 10 HEPES, 0.1 EGTA, 5 MgATP, 5 creatine phosphate, and 0.05 cAMP adjusted to pH 7.2.

### Measurement of intracellular pH (pH_i_)

Temporal intracellular H^+^ was measured using a membrane-permeant acetoxymethyl ester form of the fluorescent H^+^-sensitive indicator, SNARF-1 AM (10 μM, 5–10 min, Molecular Probes, Eugene, OR). All experiments were performed at 37 °C. In some experiments, pH buffer capacity was increased with NaHCO_3_ (25 mM) + CO_2_ (5 %) instead of HEPES in the perfusion solution to examine the effect of pH on myocyte responses to NF.

#### Mathematical modeling

The endocardial myocyte model by Pandit et al. [17] was employed to simulate the effects of fatty acids on electrophysiology and Ca^2+^ handling of rat ventricular myocytes.

The effects of intracellular acidosis induced by fatty acids [12] on cardiac ion channels and Ca^2+^ handling proteins were adopted from the results by Saegusa et al. [22]. In their report, the simulated effects of intracellular acidosis (~0.5 U) on I_CaL_ were 24 % reduction in the maximum conductance, 9 mV leftward shift of activation, 3.5 mV leftward shift of inactivation, and 20 % slowing of inactivation. The simulated effects of intracellular acidosis on other cardiac ion channels and transporters were 50 % reduction in maximum conductance of I_to_, 20 % reduction in maximum conductance of I_NaCa_, 40 % reduction in maximum Ca^2+^ flux through RyR channel, and 40 % reduction in maximum pumping rate of sarcoplasmic reticulum Ca^2+^-ATPase (SERCA), respectively. As we assumed the change in intracellular pH to be ~0.1 U, all the changes were scaled down to 1/5 of those from Saegusa et al. [22] and are summarized in Table 1. The effects of fatty acids on myofilament Ca^2+^-sensitivity were simulated by reducing Ca^2+^ on rates for troponin high- and low-affinity sites (see Table 1).

**Table 1:** Model parameters for simulation of the effects of NF on membrane currents and sarcoplasmic reticulum function

Parameter	Unit	Description	NT	NF
g_CaL_	nS	Maximum conductance for I_CaL_	31.000	29.512
V_h,act,CaL_	mV	Half-activation voltage for I_CaL_	−15.3	−17.1
k_act,CaL_	mV	Slope factor of activation for I_CaL_	5.0	4.8
V_h,inact,CaL_	mV	Half-inactivation voltage for I_CaL_	−26.7	−27.4
SF_tau,inact,CaL_	–	Scaling factor for inactivation τ of I_CaL_	1.00	1.04
g_to_	nS	Maximum conductance of I_to_	569.2575	512.3318
k_NaCa_	mM^−4^	Scaling factor for I_NaCa_	9.984·10^−3^	9.584·10^−3^
ν_1_	ms^−1^	Maximum RyR (ryanodine) channel Ca^2+^ flux	1.800	1.656
K_SR_	–	Scaling factor for Ca^2+^-ATPase	1.00	0.92
${K}_{\mathrm{htrpn}}^{+}$	mM^−1^ s^−1^	Ca^2+^ on rate for troponin high-affinity sites	200	20
${K}_{\mathrm{ltrpn}}^{+}$	mM^−1^ s^−1^	Ca^2+^ on rate for troponin low-affinity sites	40	4

See glossary of Pandit’s model [17] for detailed explanation. All other model parameters are the same as those of endocardial myocyte in the Pandit’s model.

### Statistics

Data were expressed as means ± S.E. or as relative to control (100 %) and *n* indicates the number of cells used. For all comparisons, cells were obtained from a minimum of three hearts per treatment group per protocol. Data were analyzed using Student’s *t* test and one-way ANOVA. A value of *P* < 0.05 was considered to be statistically significant.

## Results

### LV myocyte contraction and relaxation with metabolic substrates’ supplementation (NF)

**Fig. 1:**
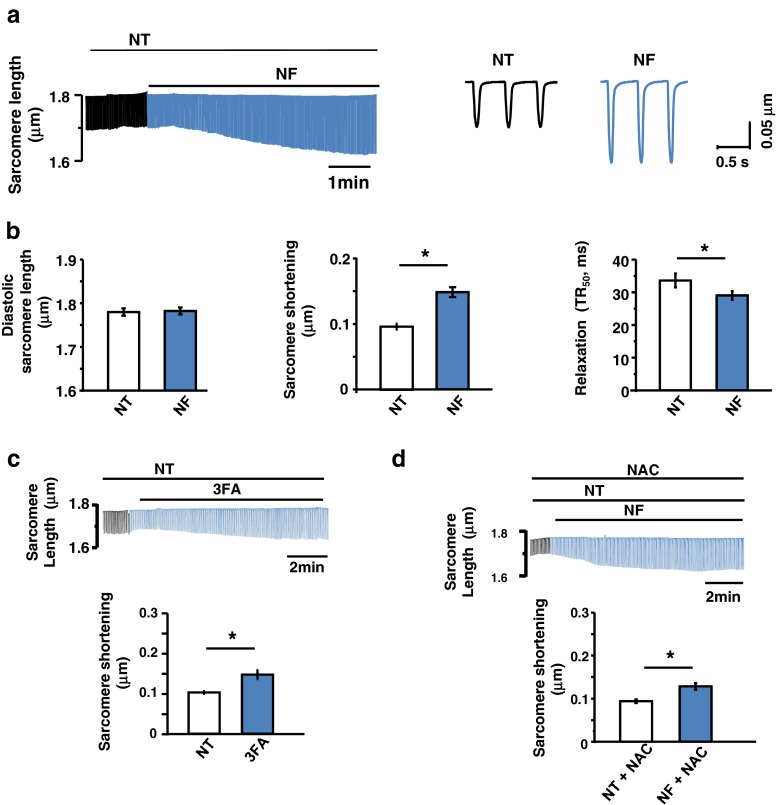
Effect of NF on LV myocyte contraction. **a** Representative raw traces of sarcomere shortening and relengthening in the presence of NF. **b** Mean values of the diastolic sarcomere length and the amplitude of sarcomere shortening (peak height) and 50 % relaxation time (TR_50_). Diastolic sarcomere length was not different between NT and NF. Sarcomere shortening was significantly increased in NF and TR_50_ was shorter by NF. **c** Representatives raw traces and mean values of three fatty acids (3FAs) only on myocyte contraction. 3FAs increased myocyte contraction. **d** Representative raw traces and mean values of a potent antioxidant NAC on myocyte contraction in NF. NAC did not affect the increment of myocyte contraction by NF

As shown in Fig. 1a, b, NF increased LV myocyte contraction (*P* < 0.001 between NT and NF, *n* = 29 & 29) and facilitated relaxation (time to 50 % relaxation, *P* < 0.02) without changing diastolic sarcomere length (*P* = 0.5). Supplementation of three types of fatty acids (linoic acid, oleic acid and palmitic acid) increased sarcomere shortening (*P* < 0.01 between NT and 3FA, *n* = 15 and *n* = 15, Fig. 1c), similar to that observed with NF. These results suggest that metabolic substrates and fatty acids increase myocyte contraction in healthy rat heart.

Since fatty acids are shown to increase intracellular reactive oxygen species (ROS) in cardiac myocytes [20, 27], possibly due to increased beta-oxidation in mitochondria and ROS potentiates myocyte contraction in murine heart [27], we tested whether ROS is responsible for the inotropic effect of metabolic substrates. Incubation of LV myocytes with a potent antioxidant, N-acetyl-cysteine (NAC, 100 μM), did not prevent NF augmentation of myocyte contraction (*P* < 0.001 between NT + NAC and NF + NAC, *n* = 16, Fig. 1d). These results exclude the role of ROS in mediating NF-induced myocyte contraction in rat hearts.

### Intracellular Ca^2+^ transient ([Ca^2+^]_i_) with NF

**Fig. 2:**
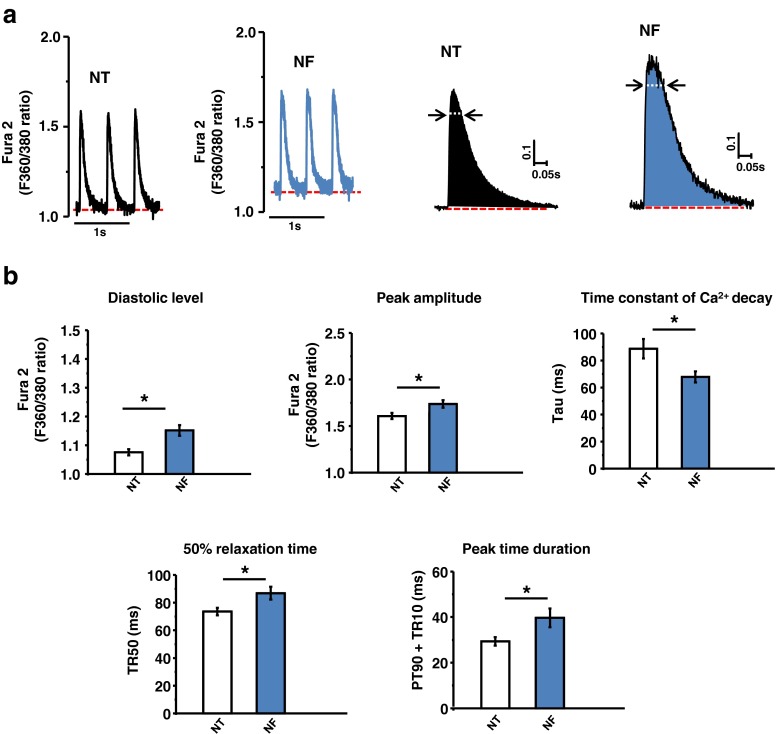
NF regulation of intracellular Ca^2+^ transients. **a** Representative [Ca^2+^]_i_ transients in NT and NF. **b** Mean values of [Ca^2+^]_i_ transient parameters. NF significantly increased diastolic [Ca^2+^]_i_ and peak amplitude of [Ca^2+^]_i_. Time to 50 % relaxation and peak time duration (PT_90_ + TR_10_) were prolonged; however, time constant of [Ca^2+^]_i_ decay (tau) was facilitated in NF

Next, we tested whether NF increased [Ca^2+^]_i_. As shown in Fig. 2a, b, NF significantly increased the diastolic and systolic [Ca^2+^]_i_ (F_360_/_380_: *P* < 0.001 and *P* < 0.001, *n* = 18, *n* = 18). Analysis of [Ca^2+^]_i_ profile showed that the time constant of [Ca^2+^]_i_ decay (tau, ms) was abbreviated in NF (*P* < 0.001, Fig. 2b). Nevertheless, the peak [Ca^2+^]_i_ duration (90 % peak time + 10 % time to relaxation, PT_90_ + TR_10_) and TR_50_ of [Ca^2+^]_i_ were prolonged (*P* = 0.009 and *P* = 0.002, *n* = 18 and *n* = 18, respectively). These results suggest that NF increased [Ca^2+^]_i_ transients and decay kinetics of [Ca^2+^]_i_ but prolonged peak time of [Ca^2+^]_i_ during systole.

### L-type Ca^2+^ current (I_Ca_) and action potential profile with NF

**Fig. 3:**
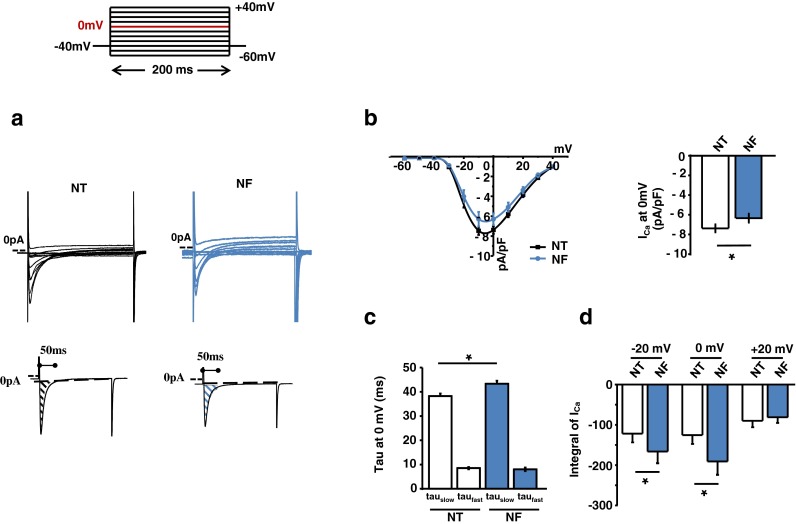
Patch-clamp recordings of LTCC activities in NF. **a**, **b** Pulse protocol, representative I_Ca_ and the corresponding I-V relationship, peak I_Ca_ at 0 mV, inactivation parameters, and the integral of Ca^2+^ influx in NF. NF reduced peak I_Ca_ density, prolonged slow inactivation of I_Ca_, and increased integral of I_Ca_ at 0 mV and −20 mV

We aimed to test the key elements of excitation-contraction coupling responsible for increased myocyte [Ca^2+^]_i_ and contraction in NF. Figure 3a, b showed raw traces and averaged current–voltage (I–V) relationships of I_Ca_ in LV myocytes before and after NF. And the inset in Fig. 3a showed raw traces of I_Ca_ at 0 mV for Ca^2+^ influx analysis. NF reduced the amplitude of I_Ca_ at 0 mV (current density of I_Ca_ at 0 mV, pA/pF: *P* = 0.03 between NT and NF, *n* = 34, *n* = 11) but slowed the inactivation of I_Ca_ at 0 mV (in ms, tau_slow_: *P* = 0.005; tau_fast_: *P* = 0.3, Fig. 3c). As a result, total Ca^2+^ influx via LTCC (integral of I_Ca_) was greater in NF (*P* = 0.05 between NT and NF at 0 mV, Fig. 3d). Similarly, integral of I_Ca_ was bigger at −20 mV (P = 0.05) but not at +20 mV.

**Fig. 4:**
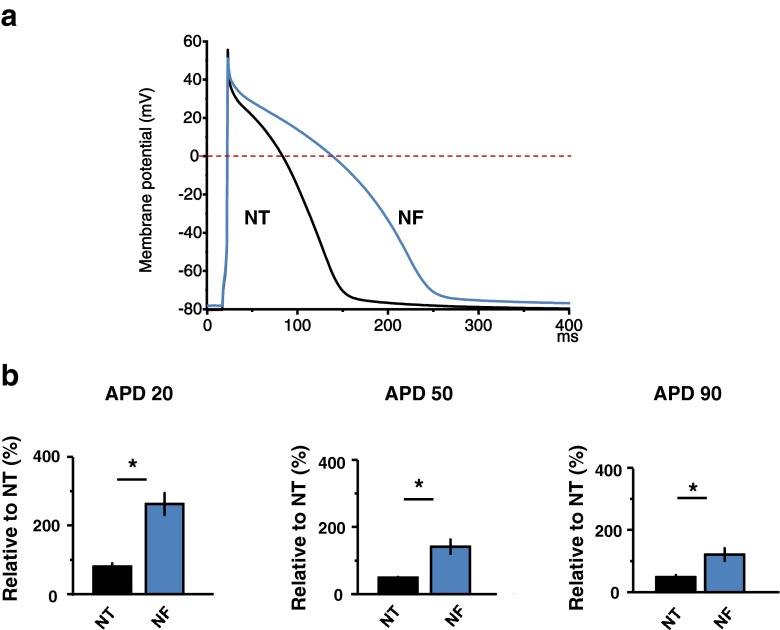
Effect of NF on action potential profile. **a** Representative action potential profile in NT and NF. **b** Mean value of action potential duration parameters. NF significantly prolonged the repolarization duration of APD (APD20, APD50, APD90)

Greater I_Ca_ influx in NF may be responsible for the prolongations of action potential (AP) plateau (and therefore APD) and [Ca^2+^]_i_ duration. Accordingly, we examined the APD duration in NF. As shown in Fig. 4a, b, APD_20_, APD_50_, and APD_90_ were significantly prolonged in NF (*P* < 0.05, *P* < 0.05, and *P* < 0.05, *n* = 12). Collectively, these results suggest that Ca^2+^ influx via LTCC is greater and APD is prolonged, which may be responsible for increased [Ca^2+^]_i_ amplitudes and prolonged peak time of [Ca^2+^]_i_ in NF.

### Myofilament Ca^2+^ sensitivity with NF

**Fig. 5:**
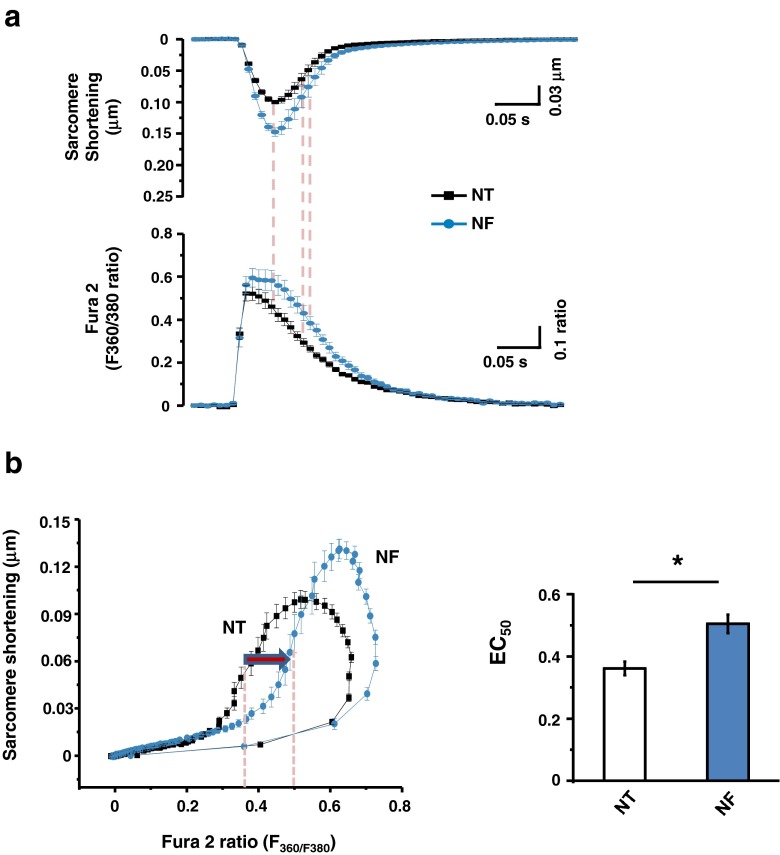
NF regulation of myofilament Ca^2+^ sensitivity. **a** Simultaneous recordings of LV myocyte sarcomere shortening/relengthening and intracellular Ca^2+^ transients in NT and in NF. **b** Phase-plane loop of sarcomere shortening/relengthening vs. [Ca^2+^]_i_ transient. The relaxation phase of the loop shifted to the right in NF compared to that in NT. Ca^2+^ concentration at 50 % sarcomere relengthening (EC_50_) was significantly larger in NF

To test whether myofilament Ca^2+^ sensitivity is regulated by NF and contributes to greater contraction and faster relaxation, we recorded sarcomere shortening and [Ca^2+^]_i_ simultaneously in Fura-2-loaded LV myocytes and plotted a phase-plane loop of the changes in sarcomere length vs. [Ca^2+^]_i_ transient with and without NF. As shown in Fig. 5a, b, relaxation phase of the sarcomere length-[Ca^2+^]_i_ transient relationship shifted to the right in NF; [Ca^2+^]_i_ at 50 % sarcomere relengthening (EC_50_) was significantly increased (*P* < 0.001, *n* = 18). These results suggest that myofilament Ca^2+^ sensitivity was significantly reduced by NF.

### Myofilament Ca^2+^ desensitization regulates [Ca^2+^]_i_ in NF

**Fig. 6:**
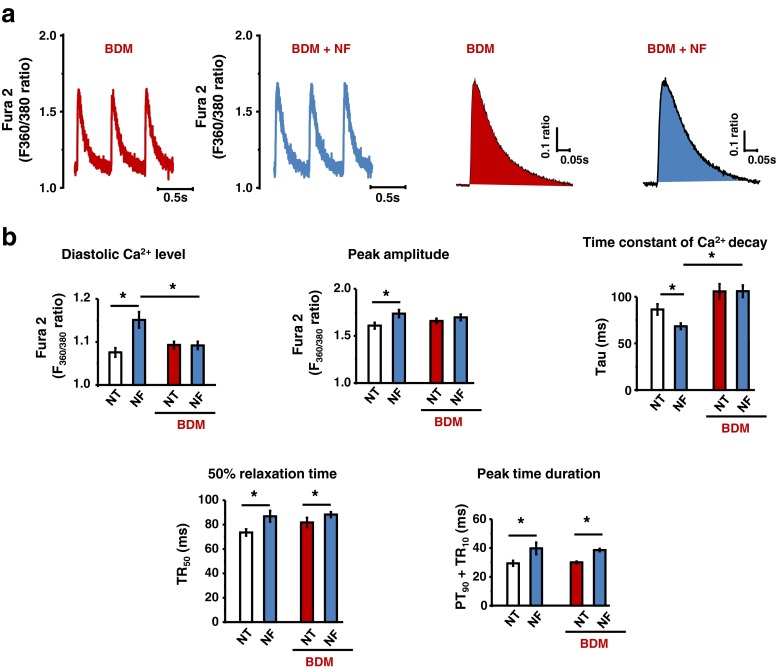
Effect of NF on [Ca^2+^]_i_ in BDM-pretreated LV myocytes. Representative [Ca^2+^]_i_ (**a**) and mean vales of [Ca^2+^]_i_ transient parameters (**b**). NF-induced increase in diastolic and systolic [Ca^2+^]_i_ were abolished by BDM (5 mM). Time constant of Ca^2+^ decay (tau) was not facilitated in NF in the presence of BDM. Peak time duration remained unaltered by BDM pretreatment

Myofilament buffers Ca^2+^ and changing myofilament Ca^2+^ sensitivity affects [Ca^2+^]_i_ homeostasis [4, 11]. Accordingly, we tested whether myofilament Ca^2+^ desensitization with a potent myosin ATPase inhibitor, BDM, prevents increased [Ca^2+^]_i_ and facilitated tau of [Ca^2+^]_i_ in NF. As shown in Fig. 6a, b, incubation of LV myocytes with BDM (5 mM) abolished NF-induced increase in diastolic and systolic [Ca^2+^]_i_ and faster tau (*P* = 0.8 in diastole; *P* = 0.22 in systole; *P* = 0.96 for tau between NT and NF with BDM; *n* = 12). These results suggest that reduced myofilament Ca^2+^sensitivity in NF may, *at least in part*, increase [Ca^2+^]_i_ and facilitate [Ca^2+^]_i_ decline in rat LV myocytes. The duration of [Ca^2+^]_i_ peak (90 % time to peak +10 % [Ca^2+^]_i_ decline, PT_90_ + TR_10_), however, remain prolonged in NF in the presence of BDM (*P* < 0.001, between NT + BDM and NF + BDM, *n* = 12, Fig. 6b).

### Regulation of intracellular pH_i_ by NF

**Fig. 7:**
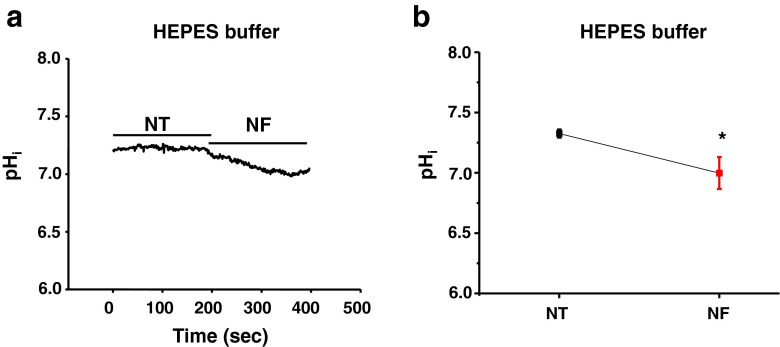
NF regulation of intracellular pH_i_. **a** Representative recording of pH_i_ in NT and NF using HEPES buffer. **b** Mean values of pH_i_ in NT and NF, pH_i_ was significantly reduced by NF

To investigate whether intracellular pH ([pH_i_]) is reduced by NF and contributes to reduced myofilament Ca^2+^ sensitivity, we detected changes of pH_i_ in SNARF-1/AM-loaded LV myocytes. As shown in Fig. 7a, the pH_i_ was gradually decreased with NF (3–5 min). Averaged results showed that NF significantly reduced pH_i_ (*P* = 0.03, *n* = 10, Fig. 7b).

Increasing pH buffer capacity in NF using NaHCO_3_ + CO_2_ (5 %) weaken the changes in pH_i_ by NF (Supplementary Fig. 1a, b). Importantly, such a modification in pH_i_ abolished NF-induced myofilament Ca^2+^ desensitization, increase in the amplitude of [Ca^2+^]_i_ and the prolongation of the peak time of [Ca^2+^]_i_ (supplementary Fig. 2a, b). These results suggest that reduced pH_i_ may play an important role in mediating the effects of NF on the myofilament Ca^2+^ sensitivity and its regulation of [Ca^2+^]_i_ handling.

### Computer simulation of I_Ca_, [Ca^2+^]_i_ and APD with NF

**Fig. 8:**
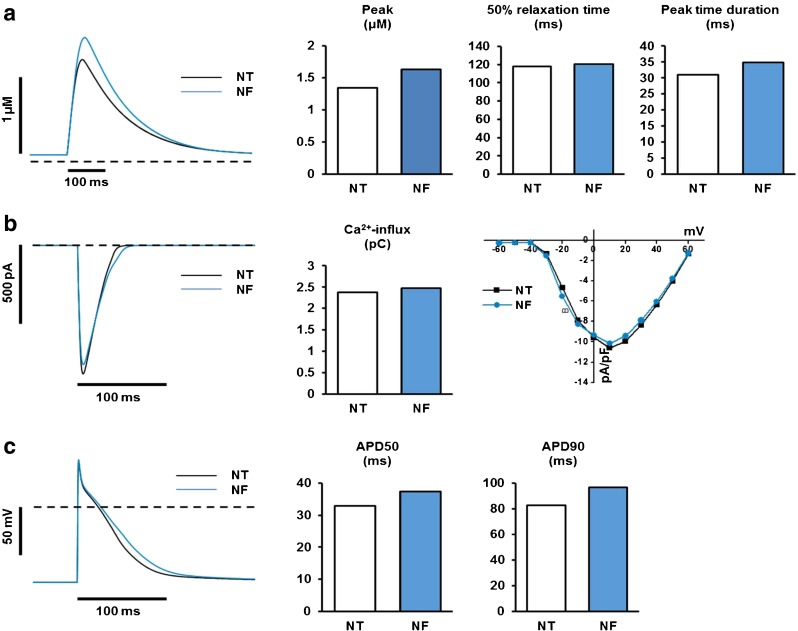
Simulated effects of NF on electrical properties and Ca^2+^ regulation of rat ventricular myocytes. Ca^2+^ transient morphology (**a**), I_CaL_ (**b**), and APD (**c**), **a** Simulated effects of NF on morphology and relaxation time of Ca^2+^ transient. Measurements of 50 % relaxation time and peak time duration were conducted in the same way as those in Fig. 2b. The *dashed line* indicates 10 nM [Ca^2+^]_i_ level. **b** Simulated effects of NF on I_CaL_ and Ca^2+^-influx through I_CaL_. The *left panel* shows the effects of NF on I_CaL_ during AP. The middle panel compares the total Ca^2+^ influx through I_CaL_ between NT and NF. The right panel shows the effects of NF on I_CaL_ density obtained by a voltage-clamp protocol used in animal experiments (see Fig. 3b). The dashed line indicates zero current level. **c** Simulated effects of NF on morphology and duration of AP. Pacing frequency is 1 Hz. APD50 was calculated as the difference between time at 50 % depolarization and time at 50 % repolarization. APD90 was calculated as the difference between time at 10 % depolarization and time at 90 % repolarization. The *dashed line* indicates zero voltage level

Next, we employed the computer simulation to further confirm whether reduced pH_i_ and concomitant myofilament Ca^2+^-desensitization affect [Ca^2+^]_i_, I_Ca_, and AP in the same way as NF. Indeed, the peak amplitude of [Ca^2+^]_i_ was increased from 1.34 to 1.63 μM, qualitatively similar to the experimental results in Fig. 2 (Fig. 8a). In addition, peak [Ca^2+^]_i_ duration was also prolonged (Fig. 8a), in line with the results observed in Fig. 2b. I–V relationship of I_Ca_ and the peak I_Ca_ at 0 mV were slightly reduced in the simulation while the integral of I_Ca_ was increased (Fig. 7b). In addition, the AP duration was prolonged in the simulation (APD_50_ from 32.8 to 37.4 ms and APD_90_ from 82.6 to 96.4 ms), which is in line with the experimental results.

## Discussion

The present study showed that supplementation of metabolic substrates (including fatty acids, physiological concentration of glucose) increased LV myocyte contraction and facilitated relaxation from rat heart by increasing Ca^2+^ influx via LTCC and increasing [Ca^2+^]_i_ transients, despite that myofilament Ca^2+^ sensitivity was reduced. Importantly, both experimental and computer simulation results suggest that myofilament Ca^2+^ desensitization, at least in part, contributes to increased [Ca^2+^]_i_ , greater contraction, abbreviated tau, and faster relaxation by NF. Intracellular pH, which is reduced by NF, is important in mediating the effects of NF on myocyte contraction, relaxation, and [Ca^2+^]_i_. These results suggest that supplementation of metabolic substrates (including fatty acids) potentiates myocyte inotropy and lusitropy by regulating key elements of Ca^2+^ handling processes and myofilament Ca^2+^ sensitivity, in addition to their effects on cardiac metabolism.

The main theme of the study was that supplementation of metabolic substrates essential for cardiac metabolism (e.g. FAs for beta-oxidation in addition to glucose, NF) influenced myocyte function from normal rat hearts. That is, NF enhanced LV myocyte contraction and promoted myocyte relaxation in almost all the myocytes studied. Furthermore, NF increased diastolic and systolic [Ca^2+^]_i_, facilitated Ca^2+^ reuptake into the SR via SERCA, and prolonged peak time duration of [Ca^2+^]_i_. Since LTCC and Ca^2+^ release from the SR are important in shaping AP and [Ca^2+^]_i_ profiles and myofilament Ca^2+^ sensitivity and myofilament mechanics dominate myocyte contraction, we analyzed these parameters in the presence of NF. As shown in both experimental and computer simulation results, peak I_Ca_ density was reduced by NF; however, the integral of I_Ca_ (total Ca^2+^ influx) was increased due to slower inactivation of LTCC (especially at the voltage range of AP plateau, −20 and 0 mV), suggesting more Ca^2+^ is fluxed into the myocyte under these conditions. Accordingly, APD was prolonged remarkably with NF. These results suggest that [Ca^2+^]_i_ elevation with NF may be due to increased Ca^2+^ influx via LTCC and the changes in LTCC are likely responsible for the prolongations of APD and the peak duration of [Ca^2+^]_i_.

NF regulation of myofilament Ca^2+^ sensitivity was analyzed in contracting LV myocytes. Our results showed a significant reduction in myofilament Ca^2+^ sensitivity with NF but myocyte contraction was increased. The seemingly counterintuitive results stress the importance of Ca^2+^ handling in greater myocyte contraction with NF. It should be noted that alterations in the myofilament Ca^2+^ sensitivity, in general, is associated with changes in Ca^2+^ binding affinity to TnC and [Ca^2+^]_i_ buffering capacity [11, 19]. For example, Robinson et al. showed that the Ca^2+^ binding affinity of the myofilament complex was reduced in dilated cardiomyopathy due to the mutations in thin filament, where myofilament Ca^2+^ sensitivity was reduced [19]. Conversely, hypertrophic cardiomyopathy with myofilament Ca^2+^-sensitizing mutant increased Ca^2+^-binding affinity and Ca^2+^ buffering [19, 21]. In fact, previous results from our laboratory have shown that myofilament Ca^2+^ desensitization in LV myocytes from angiotensin II-induced hypertensive rat hearts was associated with greater [Ca^2+^]_i_ [13] and myofilament Ca^2+^ desensitization with BDM increased [Ca^2+^]_i_ in sham but not in hypertension (where myofilament Ca^2+^ sensitivity was reduced). Furthermore, myofilament Ca^2+^ desensitization in hypertension, with BDM or with high stimulation frequency, prompted greater I_Ca_ inhibition due to [Ca^2+^]_i_ increment [25]. Here, the current findings that myofilament Ca^2+^ desensitization with BDM prevented NF-induced increase in the amplitude of [Ca^2+^]_i_ are consistent to our previous results. Furthermore, computer simulation with reduced Ca^2+^ binding and myofilament Ca^2+^ desensitization recapitulated greater [Ca^2+^]_i_ and the prolongation of the peak duration of [Ca^2+^]_i_, supporting the experimental results with NF. These results strongly suggest that both LTCC and myofilament contribute to increased [Ca^2+^]_i_ and myocyte contraction/relaxation with NF in rat heart.

Multiple mechanisms may underlie the regulation of myofilament Ca^2+^ sensitivity and LTCC by NF. It is possible that enhanced mitochondrial activity secondary to the supplementation of metabolic substrates may produce greater ROS via oxidative phosphorylation [15] and ROS can potentiate myocyte contraction via the activation of second messengers including protein kinase A (PKA) [6, 27]. As such, we tested whether intracellular ROS is involved in greater myocyte contraction with NF. Although we did not detect the ROS production from LV myocytes with NF, preincubation with a potent antioxidant, NAC failed to prevent the effect of NF on myocyte shortening. Furthermore, inhibition of PKA (with membrane permeable PKA inhibitor, PKA-amide 14-22) failed to affect NF regulation of myocyte contraction (data not shown). These results excluded the involvement of ROS in mediating the effect of NF.

Our results showed that intracellular pH was reduced by NF. Since low pH is a potent modulator of myofilament Ca^2+^ sensitivity in cardiac and skeletal muscle [1, 9, 16, 24], reduced pH may mediate the reduced myofilament Ca^2+^ sensitivity and the subsequent regulation of [Ca^2+^]_i_. To confirm, when intracellular pH buffering was increased with NaHCO_3_ + CO_2_, the effects of NF on myofilament Ca^2+^ sensitivity and [Ca^2+^]_i_ were diminished. Alternatively, reduced pH may increase [Ca^2+^]_i_ via enhancing the activity of Na^+^-H^+^ exchanger and the reverse mode of cardiac Na^+^-Ca^2+^ exchanger [5]. It is possible that increased cellular metabolic status in NF reduced intracellular pH. Alternatively, it is well established that pyruvate or lactate enters the myocyte with one proton through the sarcolemmal monocarboxylate-proton symporter [7] and consequently reduces intracellular pH [18]. Mechanism leading to the changes in intracellular pH needs further investigation.

Another possible mechanism for myofilament Ca^2+^ desensitization is intracellular ATP. This is because ATP desensitizes myofilament response to Ca^2+^ in muscle and myocardium [3, 10]. Preliminary results showed that cellular ATP level tended be elevated in normal rat LV myocyte homogenates preincubated with palmitic acid (30 min) (data not shown). The level of ATP was not detected with NF due to the lack of possibility for real-time temporal measurement in the current experimental setting. The details of myofilament Ca^2+^ desensitization and myofilament buffering of Ca^2+^ with metabolic substrates need further investigation.

### Simulated effects of NF on Ca^2+^ transients, I_CaL_, and AP

Most of experimentally observed effects of NF on AP, Ca^2+^ transients, and I_Ca_ were successfully reproduced by computer simulation which assumed that the effects of NF are all mediated by lowered intracellular pH. Saegusa et al. [22] proposed that the increased H^+^ concentration at low intracellular pH affects I_Ca_ by modulating charge screening/binding of surface charge and permeability of LTCC. As NF was found to reduce intracellular pH ~0.1 U (Fig. 8a) in our experimental condition, modifications proposed by Saegusa et al. [22] were all scaled down to 1/5 and were applied to our simulation. The increased intracellular H^+^ is also known to raise intracellular Ca^2+^ by unloading Ca^2+^ from Ca^2+^-buffering proteins (coupled Ca^2+^/H^+^ transport by cytoplasmic buffers regulates local Ca^2+^ and H^+^ ion signaling [23]). In our simulation, the ability of increased intracellular H^+^ to unload Ca^2+^ from Ca^2+^-buffering proteins was embodied by assuming it reduces Ca^2+^ on rates for TnC. Therefore, the effects of NF were reproduced in our simulation by assuming that L-type Ca^2+^ channels and Ca^2+^-buffering are both affected in response to increased intracellular H^+^. The altered LTCC was found to be responsible for both increased amplitude of Ca^2+^ transients and the prolongation of AP in our simulation, whereas the altered Ca^2+^-buffering was found to be mainly responsible for increased amplitude of Ca^2+^ transients rather than prolongation of AP (data not shown).

In conclusion, our results clearly show that supplementation of metabolic substrates, including fatty acids, is important in strengthening cardiac metabolism and contractile function. Under these conditions, Ca^2+^ handling processes and myofilament play in concert to potentiate intracellular Ca^2+^ level and induce greater contraction. Our results provide useful evidence for better understanding of cardiac excitation–contraction coupling with comprehensive metabolic supplies.

## Electronic supplementary material

Supplementary Figure 1 (PDF 17.5 kb)

Supplementary Figure 2 (PDF 161 kb)
