# Supplementary figures and images for: Cardiac inotropy, lusitropy, and Ca2+ handling with major metabolic substrates in rat heart
Source: Pflugers Arch. 2016 Oct 28;468(11):1995–2006. doi: 10.1007/s00424-016-1892-8 (PMC5138277; doi:10.1007/s00424-016-1892-8)

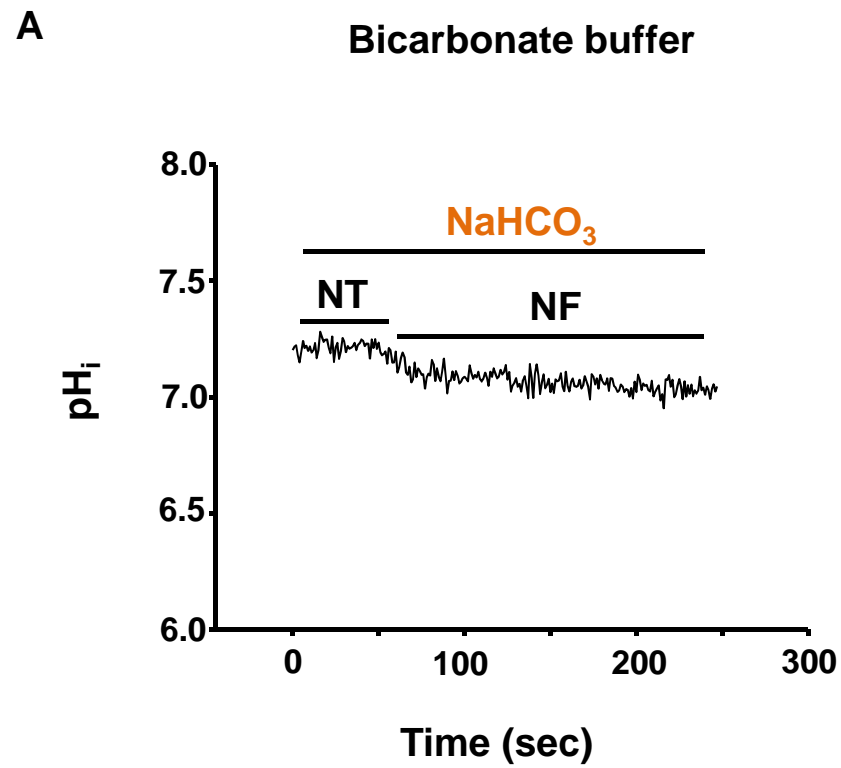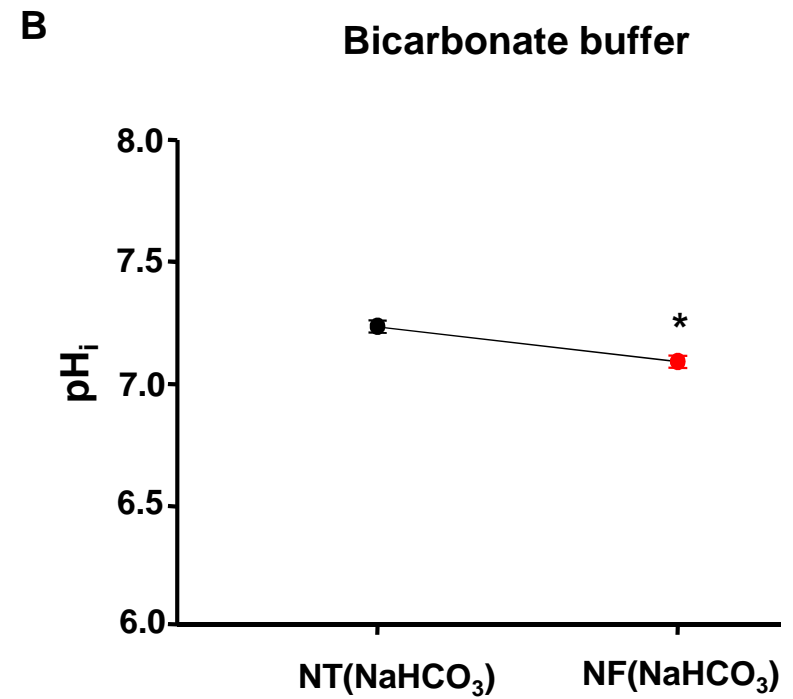

Supplementary figure 1

Supplement: Supplementary file 1 — (PDF 17.5 kb) [file 424_2016_1892_MOESM1_ESM.pdf]

**A**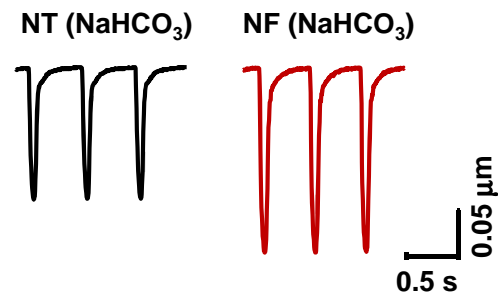**B**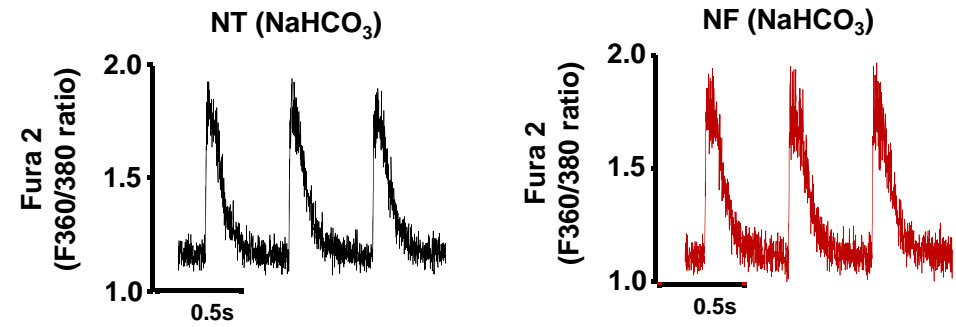**C**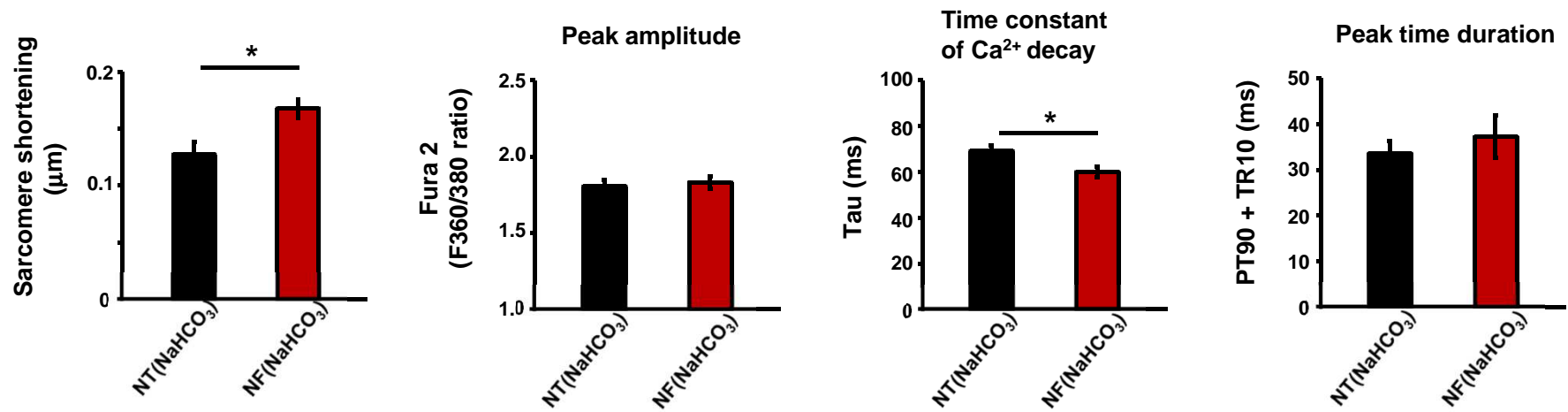**D**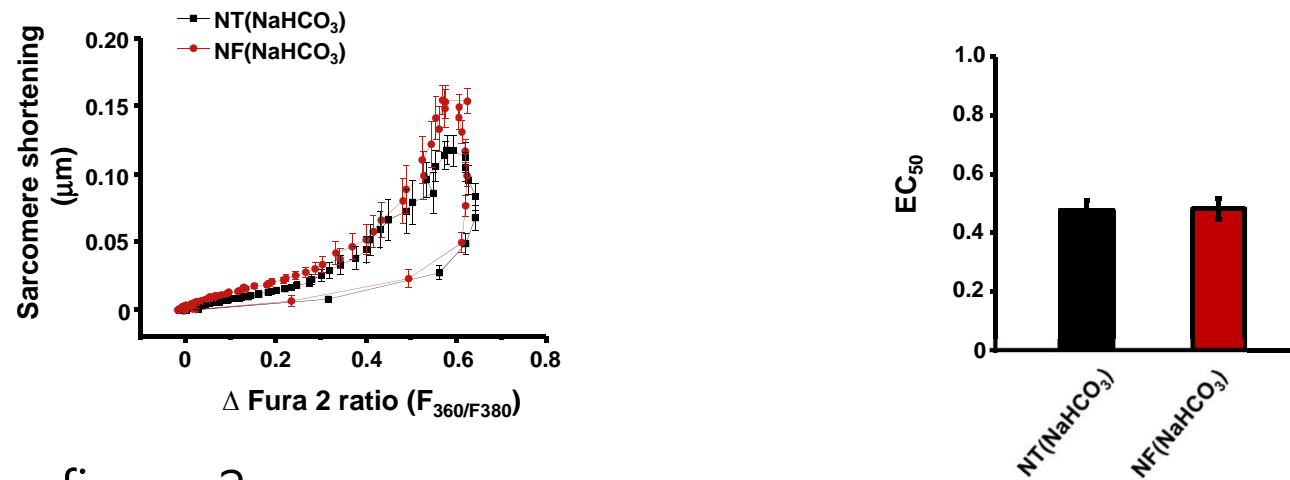

Supplementary figure 2

Supplement: Supplementary file 2 — (PDF 161 kb) [file 424_2016_1892_MOESM2_ESM.pdf]
